# Septo-optic dysplasia with amniotic band syndrome sequence: a case report

**DOI:** 10.1186/s13256-019-2306-2

**Published:** 2019-12-16

**Authors:** Insiyah A. Amiji, Ummulkheir H. Mohamed, Adelina G. Rutashobya, Mariam Mngoya, Nicole Schoenmann, Helga E. Naburi, Karim P. Manji

**Affiliations:** 1 Department of Paediatric and Child Health, School of Medicine, Muhimbili University of Health and Allied Sciences, PO Box 65001, Dar es Salaam, Tanzania; 2 Asklepios Klinik Barmbek, PO Box 22307, Hamburg, Germany

**Keywords:** Septo-optic dysplasia, Amniotic band syndrome, Optic nerve hypoplasia, Hypopituitarism

## Abstract

**Introduction:**

De Morsier syndrome, or septo-optic dysplasia, is a rare, heterogeneous, complex condition with a highly variable phenotype. It is characterized by optic nerve hypoplasia, pituitary gland hypoplasia, and midline brain abnormalities, including absence of septum pellucidum and corpus callosum dysgenesis. Diagnosis is made clinically by the presence of any two or more features from the clinical triad.

**Case presentation:**

We report a case of a premature African newborn male baby born to nonconsanguineous parents who presented to our institution with agenesis of the septum pellucidum, unilateral optic nerve hypoplasia, and pituitary stalk hypoplasia. However, he had intact central endocrine function. He also presented with limb defects due to constricting amniotic band syndrome. Other dysmorphic features were low-set ears, microcephaly, and bilateral talipes equinovarus. He otherwise had a normal neurological examination result. Over time, he had an adequate weight gain and was managed by a multidisciplinary team.

**Conclusion:**

De Morsier syndrome still represents a diagnostic challenge, despite advances in neuroimaging and genetic studies, due to the heterogeneous nature of the disorder. This case adds to existing knowledge on the vascular pathogenesis of septo-optic dysplasia.

## Introduction

Septo-optic dysplasia (SOD) is a rare heterogenous condition comprising optic nerve hypoplasia, midline brain abnormalities (agenesis of corpus callosum or absence of septum pellucidum), and pituitary hypoplasia with consequent hypopituitarism.

The majority of cases are sporadic; however, several familial cases have been described, and the identification of mutations in key developmental genes, including HESX1, SOX2, and SOX3, suggests a genetic causation. Therefore, it is a developmental disorder resulting from a defect of normal embryological development. The precise etiology is multifactorial, involving contributions from environmental factors and genetics [1].

The diagnosis is predominantly clinical, and it is made on the basis of the presence of two or more features of the classic triad: hypopituitarism, optic nerve hypoplasia, and midline brain defects, typically absence or hypoplasia of the septum pellucidum and/or corpus callosum.

There is evidence in the literature of the vascular pathogenesis of SOD [2]. However, the mechanisms of congenital constriction band syndrome with SOD are still under debate. Theories have been postulated that septo-optic “dysplasia” makes more sense as a vascular disruption sequence than as a primary developmental anomaly.

## Case presentation

We report a case of a newborn African male baby delivered at 32 weeks of gestation by cesarean section due to breech presentation. His birth weight was 1.9 kg (75th percentile), head circumference was 27 cm (< 10th percentile), and length was 37 cm (< 10th percentile). He was the first child of nonconsanguineous parents. His prenatal history was uneventful. The family had no history of visual abnormalities or neurological disorders.

**Fig. 1:**
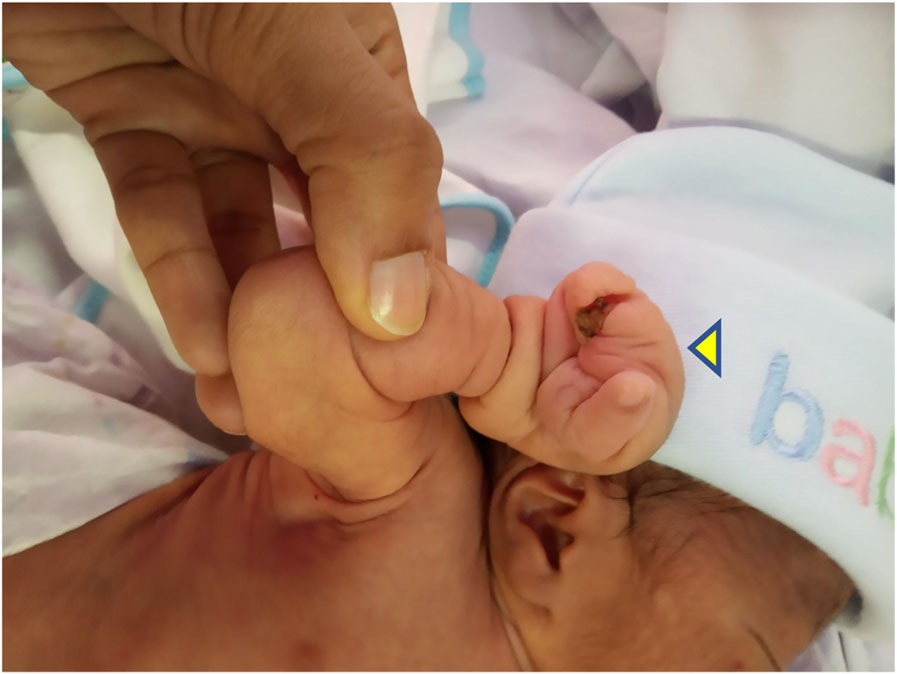
Left wrist after amniotic band release (arrowhead)

**Fig. 2:**
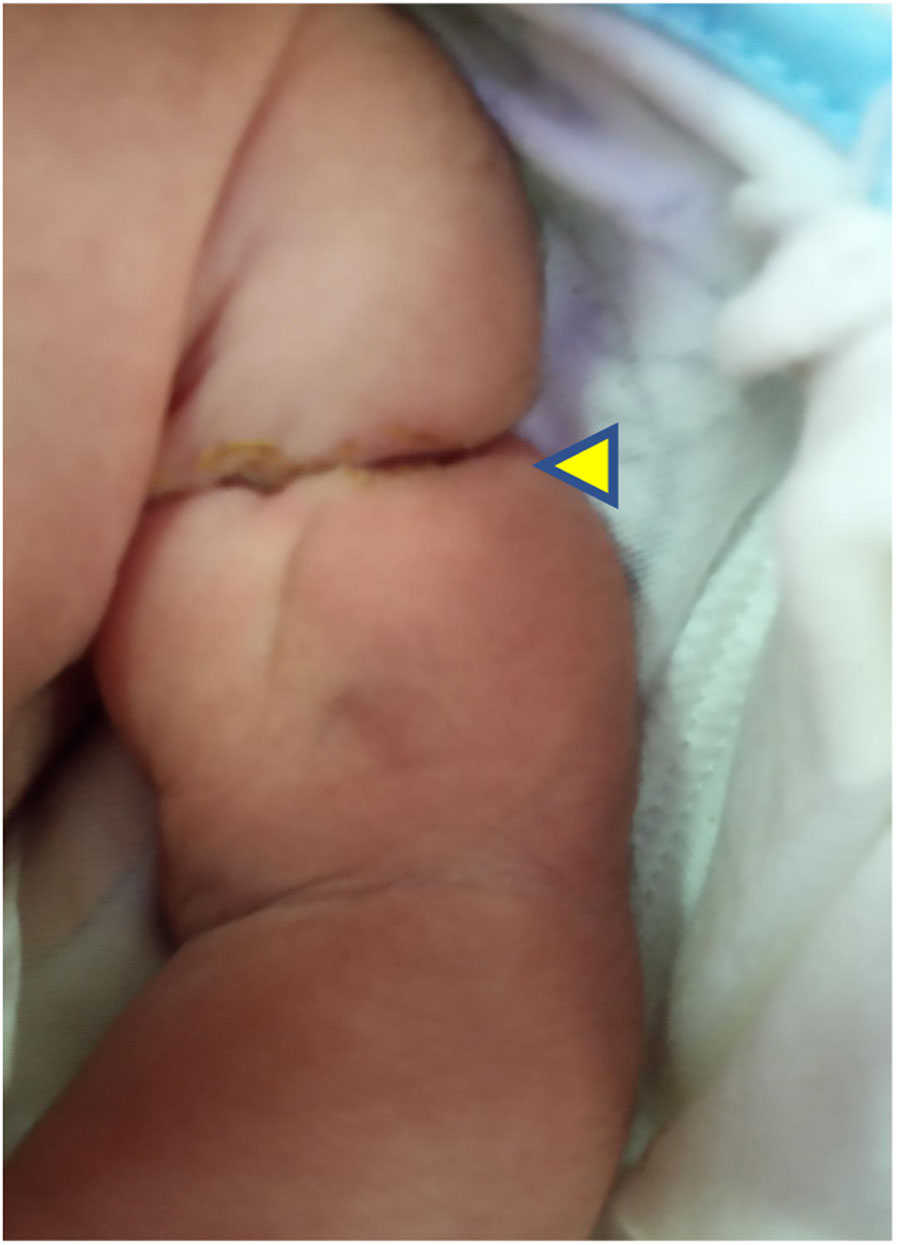
Constricting amniotic band on right arm (arrowhead)

**Fig. 3:**
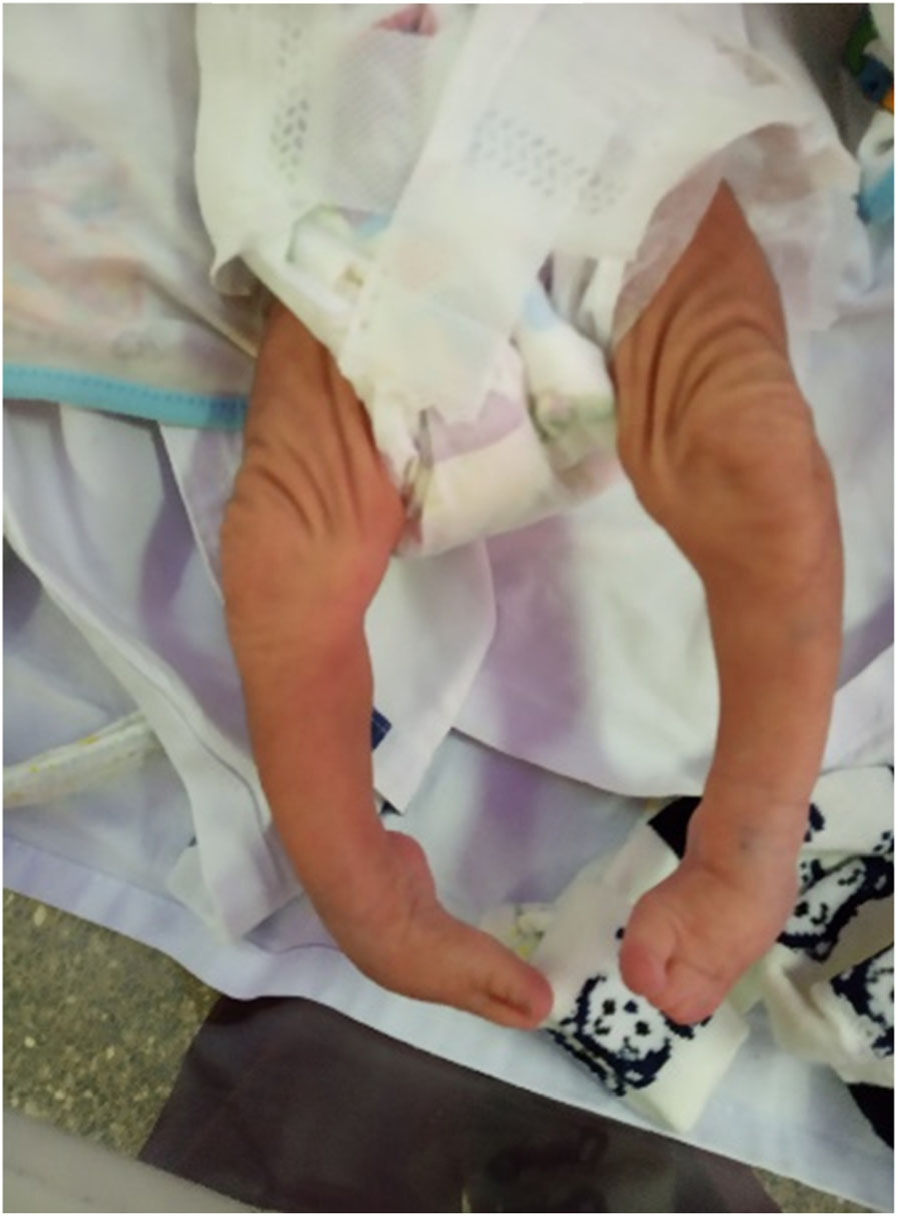
Bilateral talipes equinovarus

General examination revealed absence of all five digits in the right hand with a constricting band in the right wrist joint (Fig. 1)*.* A similar constricting band was found on the left arm (Fig. 2). Radial and brachial pulses were palpable and of good volume in both upper limbs, and they were synchronous with femoral and popliteal pulses of the lower limbs. The skin of all the limbs was normal in color. Other distinctive features were the presence low-set ears, microcephaly and bilateral talipes equinovarus (Fig. 3)*.* His random blood sugars were within normal range, and he maintained normal body temperature. He gained 600 g by the end of 3 weeks and did not experience any seizures or jitteriness throughout his stay in the hospital.

Neurological examination revealed good muscular tone and normal primitive reflexes, and both pupils had a normal reaction to light. Indirect funduscopy showed a normal optic disk in the right eye but optic nerve hypoplasia in the left eye with retinopathy of prematurity stage II.

The patient’s serum level of thyroid-stimulating hormone was 0.59 nmol/L (normal), growth hormone level was 8.27 ng/ml (high), and cortisol level was 8.1 μg/dl (normal).

**Fig. 4:**
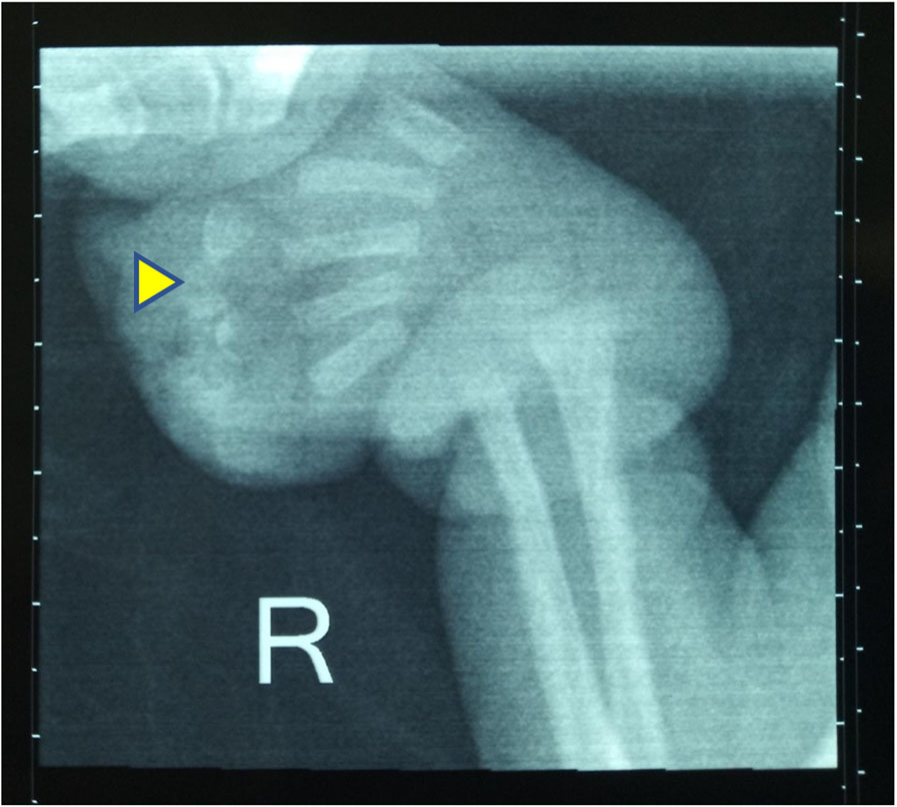
Hypoplasia of the right proximal and distal phalanges (arrowhead)

**Fig. 5:**
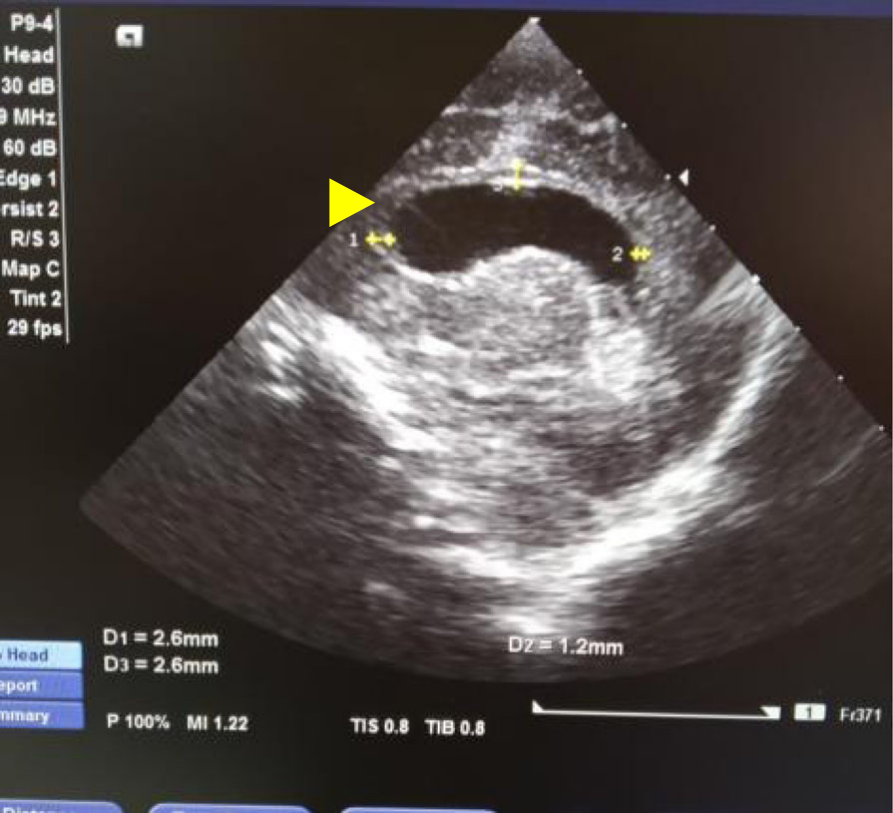
Thin corpus callosum (arrowhead)

X-ray of the right hand showed hypoplasia of proximal and distal phalanges (Fig. 4)*.* Cranial ultrasound showed agenesis of the septum pellucidum and dilation of the third ventricle (Fig. 5)*.*

**Fig. 6:**
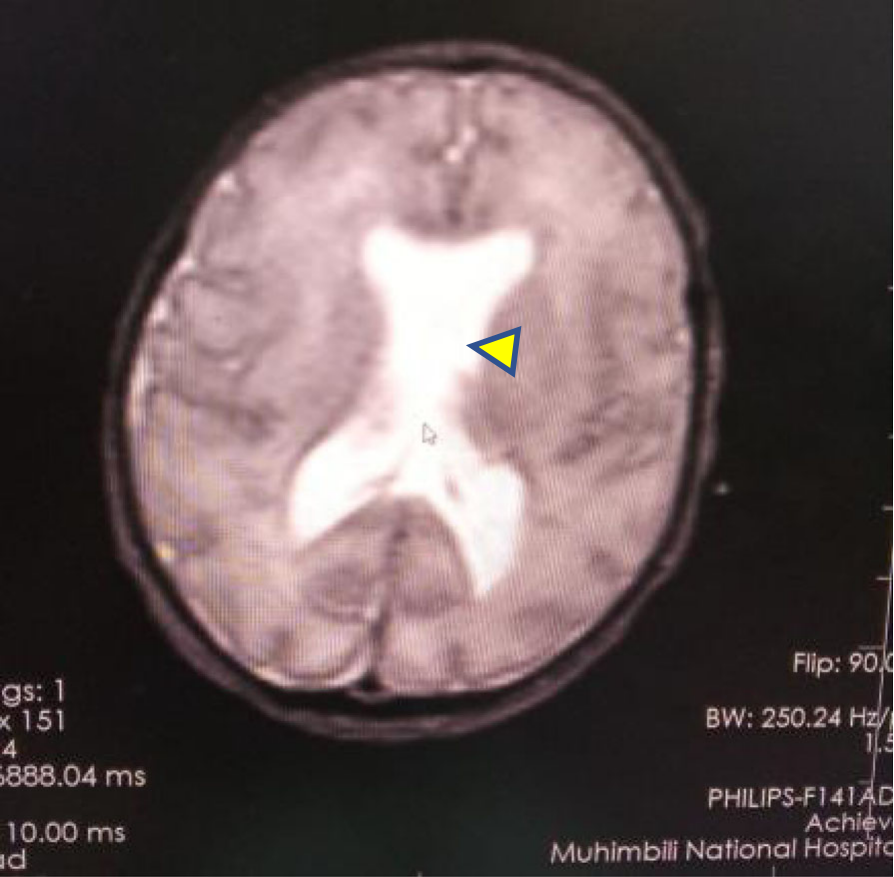
Absent septum pellucidum (arrowhead)

**Fig. 7:**
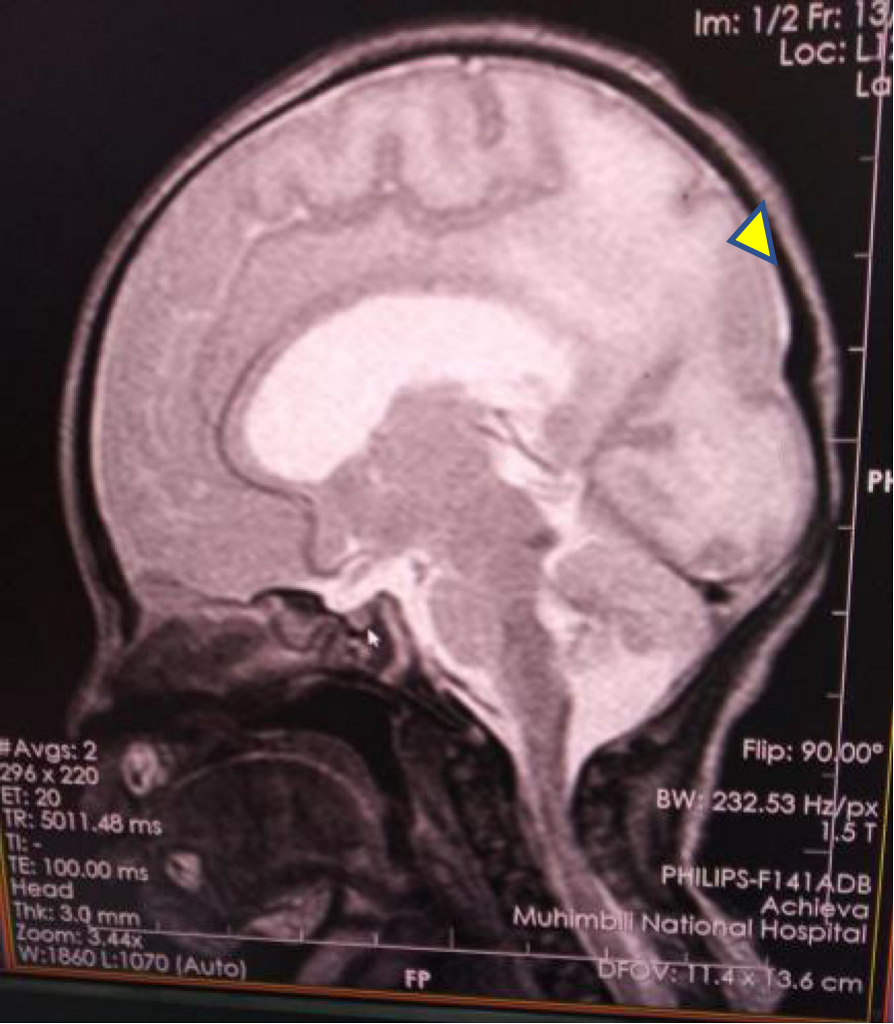
Pachygyria (arrowhead)

Magnetic resonance imaging of the brain revealed absence of the septum pellucidum, thin corpus callosum, hypoplastic pituitary stalk, bilateral blunted roof of the anterior horns of the lateral ventricles, and pachygyria. No schizencephaly was present (Figs. 6 and 7)*.*

We could not do genetic testing, because it is not available in our setting.

The child was managed conservatively with a multidisciplinary approach including a team of pediatricians, pediatric surgeons, and an ophthalmologist. The duration of his hospital stay was 3 weeks. During this time, he underwent constricting amniotic band release repair of both upper limbs, and corrective repair of the right hand was planned with plastic surgery. Currently, the patient is discharged, he is followed up as an outpatient in Opthalmology clinic every month. Because he does not have endocrinologic abnormalities at this stage, we plan to do a repeat hormone profile at the age of 6 months. Due to early detection of the abnormalities and interventions and with the cooperation of the parents, our patient has a favorable prognosis, which will improve his quality of life and survival.

## Discussion

The term “septo-optic dysplasia” was coined in 1956 by de Morsier, who pointed out the association of optic nerve hypoplasia with an absence of the septum pellucidum [3]. Later, in 1970, Hoyt *et al.* demonstrated the association between SOD and pituitary dwarfism [4]. SOD has been used to describe a variety of clinical conditions in patients with bilateral optic nerve hypoplasia and a spectrum of midline brain defects with or without endocrinologic abnormalities (Table 1).

**Table 1:** Clinical pattern of septo-optic dysplasia

Features	Our patient	Palui *et al*., 2018 [13]	Ganesh *et al*., 2013 [6]	Signorini *et al*., 2012 [5]	Polizzi *et al*., 2006 [7]	Stevens *et al*., 2004 [10]
Race (children)	African	Indian	Omani	Italian	Italian	American
Neurological						
Midline defects						
Septum pellucidum/corpus callosum						
Normal	–	–	–	–	–	–
Absent/agenesis	+	+	+	+	+	–
Cerebral cortex						
Normal	–	+	+	+	+	–
Polymicrogyria	–	–	–	+	–	+
Schizencephaly	–	–	–	+	–	–
Pachygyria	+	–	–	–	–	–
Other						
Dysmorphic fornices	–	–	–	+	+	–
Ventricular abnormality	+	–	–	+	–	–
Cerebellar vermis hypoplasia	–	–	–	+	–	–
White matter abnormalities	–	–	–	–	–	–
Ocular						
Optic nerve						
Unilateral hypoplasia	+	–	+	–	–	–
Bilateral hypoplasia	–	+	+	+	+	+
Endocrine						
Pituitary gland						
Normal	+	–	–	–	–	Not reported
Absent	–	–	–	+	–	
Ectopic	–	+	+	–	+	
Pituitary stalk						
Normal	–	+	–	+	Not reported	Not reported
Hypoplasia	+	–	+	–		
Pituitary hormones						
Normal	+	–	–	–	+	–
Low or absent	–	+	+	+	+	+
Additional findings						
Limb defects	+	–	–	–	+	+
Dysmorphic features (cleft palate, cleft lip, frontal bossing, macrocephaly, microcephaly, genital abnormalities)	+	–	–	–	+	–

Diagnosis is predominantly clinical; however, despite advances in neuroimaging and genetic studies, it still poses a diagnostic challenge due to its highly variable clinical spectrum. Our patient was diagnosed clinically and with evidence of neuroimaging after birth, but most affected children present later in life with failure to thrive, hypoglycemia, seizures, blindness, and developmental delay.

This syndrome has been linked to young maternal age, and it has been reported to be equivalent in males and females. To the best of our knowledge, this is the first case reported in an African male patient, born to nonconsanguineous parents. Male predominance has also been shown in Italian and Omani children [5, 6]. Most cases reported have no history of consanguinity, possibly indicating a sporadic cause for this disorder. In contrast, Ganesh et al. [6] reported the presence of highly prevalent consanguinity in Omani children with this disorder. Therefore, this does not exclude the possibility of a genetic cause.

SOD is a heterogenous, complex malformation with high phenotypic variability; hence, all features might not be present in a single patient [5, 7].

Full-blown de Morsier syndrome (SOD with panhypopituitarism) is seen in only in 11–30% of fully evaluated patients [8, 9]. Hypothalamic-pituitary dysfunction is a predominant feature in SOD. It manifests as either structural malformation in the pituitary gland detected by high-resolution neuroimaging or pituitary hormone insufficiencies; however, only 60% show structural abnormalities in the brain.

Our patient had midline brain defects such as agenesis of septum pellucidum, thinning of the corpus callosum, unilateral optic nerve hypoplasia, and pituitary stalk hypoplasia with intact central endocrine function, and our patient showed findings similar to those in the cases reported by Signorini *et al.* [5] in Italian children. In view of these findings and the possibility that hormone deficiencies can emerge and evolve over time, lifelong medical follow-up of these aspects may be warranted.

Theories on the vascular etiology of septo-optic “dysplasia” have been postulated that it is more a vascular disruption sequence than a primary developmental anomaly [2, 10]. The anatomic correlation of the anterior cerebral artery and its branches to the optic nerves and chiasm, the anterior hypothalamus, the septum pellucidum, and other structures makes it more vulnerable to vascular events.

The limb defects in our patient as well as those previously reported are suggestive of amniotic band disruption sequence [8, 10, 11]. We propose that these vascular changes may also explain the development of features of SOD seen in these patients.

SOD has been reported to occur with exposure to several teratogens, some of which are known to have vascular effects, including cocaine, alcohol, and phenylpropanolamine [12]. However, we found no such exposure during the prenatal period in our patient.

On the basis of the clinical findings and evidence in the literature, our patient presented with features suggestive of SOD with limb defects due to constricting amniotic band sequence, indicating a possibility of vascular pathogenesis of this syndrome.

## Conclusion

De Morsier syndrome (SOD) still represents a diagnostic challenge. Standard diagnostic criteria together with a high index of suspicion in the evaluation of patients with midline defects, optic nerve hypoplasia, and/or hypopituitarism is important for accurate detection. Furthermore, identification of different phenotypic profiles within the disease spectrum can allow more targeted follow-up of these children to reduce disease-related morbidity and mortality, which may prevent life-threatening sequelae. This case adds to the existing knowledge on the vascular etiology of SOD.

## Data Availability

Data sharing not applicable.

## Abbreviations

SOD: Septo-optic dysplasia
